# Characteristics of Anal Fistula Cancer in Patients Who Underwent Surgery: A Nationwide Observational Study Using Diagnosis Procedure Combination Data

**DOI:** 10.1002/ags3.70228

**Published:** 2026-05-05

**Authors:** Nobuaki Hoshino, Hiromitsu Kinoshita, Kazutaka Obama, Kiyohide Fushimi, Susumu Kunisawa, Yuichi Imanaka

**Affiliations:** ^1^ Department of Surgery Kyoto University Graduate School of Medicine Kyoto Japan; ^2^ Department of Health Policy and Informatics Institute of Science Tokyo Graduate School of Medical and Dental Sciences Tokyo Japan; ^3^ Department of Healthcare Economics and Quality Management, School of Public Health, Graduate School of Medicine Kyoto University Kyoto Japan; ^4^ Department of Health Security System, Center for Health Security, Graduate School of Medicine Kyoto University Kyoto Japan

**Keywords:** anal fistula, anal neoplasms, complication, medical cost

## Abstract

**Aim:**

Anal fistula cancer is a rare disease that occurs in < 1% of patients with anal fistulas. The clinical characteristics of anal fistula cancer are not fully understood. This study aimed to identify the characteristics of patients with anal fistula cancer by comparing them with those of patients with anal canal or rectal cancer.

**Methods:**

This was a nationwide observational study using the Diagnosis Procedure Combination data. Patients who underwent abdominoperineal resection or total pelvic exenteration for anal fistula, anal canal, or rectal cancer were included in this study. Patient background, surgical outcomes, postoperative complications, and medical costs were compared among the three types of cancer.

**Results:**

This study included 206 patients with anal fistula cancer, 1207 patients with anal canal cancer, and 18 254 patients with rectal cancer. Patients with anal fistula cancer were more likely to be male, younger, or smokers. In patients with anal fistula cancer, the proportion of open surgeries was significantly higher, and the length of hospital stay was significantly longer than in those with anal canal and rectal cancer. Anal fistula cancer tended to be more locally advanced than other cancers, whereas lymph node and distant metastases were less common. The incidence of incisional wound infection and wound dehiscence was significantly higher, and medical costs were the highest in patients with anal fistula cancer.

**Conclusion:**

Anal fistula cancer is characterized by aggressive local progression, resulting in a higher frequency of open surgery, a higher incidence of wound‐related complications, and higher medical costs.

## Graphical abstract

The characteristics of patients with anal fistula cancer included a higher proportion of men, younger age, more smokers, and tumor‐related characteristics, including more advanced local progression. Surgical characteristics included a lower rate of minimally invasive surgery, a higher proportion of total pelvic exenterations, and longer postoperative hospitalizations. Regarding postoperative complications, there was a higher incidence of surgical site infections, wound dehiscence, and sepsis, as well as higher medical costs.

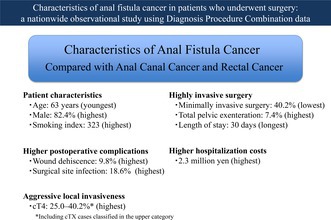

## 1 Introduction

Anal fistula cancer is thought to develop when chronic inflammation from an anal fistula persistently stimulates the anal glands and epithelial components within the fistula tract over a long period, leading to cancerous changes [1]. Anal fistula cancer is suspected in cases where the fistula has persisted for over 10 years, there is pain and induration at the site, mucous discharge is present, the fistula opening is located in the anal canal or crypt, and there are no other cancers in the rectoanal area [1, 2]. Anal fistula cancer is a rare disease that occurs in less than 1% of patients with anal fistulas [3, 4, 5]. It accounts for less than 1% of all colorectal cancers [6, 7, 8, 9]. The clinical and pathological characteristics of anal fistula cancer are not fully understood and are often detected at advanced stages [3, 4, 7, 10]. Most of the knowledge accumulated thus far on anal fistula cancer comes from case reports or studies from single institutions with small numbers of patients, and there are no systematic epidemiological data [11, 12] As cancer often arises during the long course of a fistula, and its incidence is very low, collecting clinical data is extremely challenging. However, because patients who develop anal fistula cancer are likely to be hospitalized and undergo surgery at large hospitals, the Diagnosis Procedure Combination (DPC) database is considered useful for clarifying the perioperative characteristics of anal fistula cancer [13, 14, 15]. Therefore, this study aimed to identify the characteristics of patients with anal fistula cancer by utilizing DPC data and comparing them with patients who underwent the same surgery for anal canal or rectal cancers.

## 2 Patients and Methods

### 2.1 Study Design and Setting

This observational study was a large‐scale epidemiological survey of anal fistula cancer conducted using the DPC database in Japan. The DPC database contains both receipt and discharge summary data. It comprised data from 82 universities and private hospitals that voluntarily participated, with over 1000 hospitals. This database includes patient information, such as age, sex, diagnosis at admission, comorbidities, TNM classification, surgical method, operative time, complications, and medical costs. Disease names are accompanied by ICD‐10 codes. For further details, please refer to the previously published reports [16, 17]. From this database, patients diagnosed with anal fistula cancer, anal canal cancer (ICD‐10 codes: C21.1, C21.8), or rectal cancer (ICD‐10 code: C20), identified using relevant DPC diagnosis fields (principal diagnosis, diagnosis leading to hospitalization, and diagnosis accounting for the greatest resource use), who underwent abdominoperineal resection or total pelvic exenteration (Japanese surgery codes: K740$, K7482, K645) between April 2016 and March 2023 were identified.

A comparative study on the clinical features, surgical outcomes, postoperative complications, and medical costs of anal fistulas, anal canals, and rectal cancers was conducted. Patient background factors, including age, sex, hypertension, diabetes, angina, myocardial infarction, cerebral hemorrhage, cerebral infarction, chronic obstructive pulmonary disease, smoking, body mass index, and TNM classification, were examined. The comorbidities of the patients were extracted from the “comorbidities at admission” section. The ICD‐10 codes used to identify the comorbidities are listed in Table S1. In addition, in patients with anal fistula cancer, the presence or absence of coexisting Crohn's Disease was confirmed (ICD‐10 codes: K500, K501, K508, K509). For surgical outcomes, the surgical approach, procedure, duration of general anesthesia, and length of hospital stay (LOS) were evaluated. Because the operation time was not included in the DPC data, general anesthesia time was used as a substitute indicator of surgical invasiveness. The postoperative complications assessed included incisional site infection, wound dehiscence, ileus/bowel obstruction, peritonitis, urinary dysfunction, urinary tract infection (UTI), sepsis, pneumonia, deep vein thrombosis, cerebrovascular disorders, hemorrhage, acute renal failure, cerebral thrombosis, acute coronary syndrome, and mortality. Complications were identified by extracting relevant disease names from “illnesses that developed after hospitalization.” The ICD‐10 codes used to identify the comorbidities are listed in Table S2. Medical costs were defined as total hospitalization costs calculated on a fee‐for‐service basis, regardless of the DPC‐based payment system.

### 2.2 Statistical Analysis

Continuous variables are presented as medians and interquartile ranges (IQR) and were tested using the Kruskal‐Wallis test. Categorical variables are presented as numbers and percentages, and were tested using Fisher's exact test. For items with > 5% missing values, a sensitivity analysis was performed under three conditions: not considering the missing values, the best‐case scenario, and the worst‐case scenario. A complete case analysis was conducted for items with < 5% missing values. All statistical analyses were performed using the R software (R Foundation for Statistical Computing, Vienna, Austria). All *p*‐values were two‐sided, and those < 0.05 were considered statistically significant.

This study was approved by the Ethics Committee of Kyoto University (ID: R0135). The need for informed consent was waived due to the anonymity of the data.

## 3 Results

### 3.1 Patient Characteristics

Overall, 204 patients with anal fistula cancer, 1067 with anal canal cancer, and 18 651 with rectal cancer were identified. Patient characteristics are shown in Table 1. Compared with patients with anal canal or rectal cancer, those with anal fistula cancer were more likely to be male, younger, and smokers. In terms of cancer characteristics, TNM staging data were more likely to be missing; however, among the available data, lymph node and distant metastases were less common in anal fistula cancer than anal canal and rectal cancers.

**TABLE 1:** Patient characteristics.

		Anal fistula cancer		Anal canal cancer		Rectal cancer	
		*n* = 204		*n* = 1067		*n* = 18 651	
		*n*	%	*n*	%	*n*	%
Age*		63	51–72	72	64–79	71	64–78
Sex	Male	168	82.4	571	53.5	12 210	65.5
	Female	36	17.6	496	46.5	6441	34.5
Hypertension		50	24.5	352	33.0	7015	37.6
Diabetes		18	8.8	67	6.3	1324	7.1
Angina		14	6.9	81	7.6	1277	6.8
Myocardial infarction		4	2.0	17	1.6	383	2.1
Cerebral bleeding		1	0.5	4	0.4	128	0.7
Cerebral infarction		10	4.9	53	5.0	1106	5.9
COPD		3	1.5	25	2.3	658	3.5
Smoking index*		323	0–885	0	0–670	175	0–800
Body mass index*		21.7	19.3–24.1	21.7	19.4–24.2	22.0	19.8–24.5
cT	T0,1	16	7.8	111	10.4	1271	6.8
	T2	32	15.7	279	26.1	3122	16.7
	T3	74	36.3	370	34.7	9163	49.1
	T4	51	25.0	253	23.7	4326	23.2
	TX	31	15.2	54	5.1	769	4.1
cN	N0	148	72.5	579	54.3	9052	48.5
	N+	35	17.2	442	41.4	8797	47.2
	NX	21	10.3	46	4.3	802	3.8
cM	M0	180	88.2	923	86.5	15 754	84.5
	M1	5	2.5	105	9.8	2181	11.7
	MX	19	9.3	39	3.7	716	3.8

Abbreviation: COPD, chronic obstructive pulmonary disease.

* Median, interquartile range.

### 3.2 Surgical Outcomes

Surgical outcomes are summarized in Table 2. Among patients with anal fistula, anal canal, and rectal cancer, there were significant differences in the proportion of patients who underwent minimally invasive surgery (58.8% vs. 74.3% vs. 71.2%, *p* < 0.001), duration of general anesthesia (568 min vs. 464 min vs. 448 min, *p* < 0.001), and LOS (30.0 days vs. 26.0 days vs. 26.0 days, *p* < 0.001). Although not statistically significant, the proportion of total pelvic exenteration was higher in patients with anal fistula cancer than in those with anal canal or rectal cancer (7.4% vs. 4.3% vs. 4.2%, *p* = 0.090).

**TABLE 2:** Surgical outcomes.

		Anal fistula cancer		Anal canal cancer		Rectal cancer		
		*n* = 204		*n* = 1067		*n* = 18 651		*p*
		*n*	%	*n*	%	*n*	%	
Approach	Open	82	40.2	274	25.7	5380	28.8	< 0.001
	Minimally invasive (Laparoscopic/Robotic)	122 (107/15)	59.8 (52.5/7.4)	793 (676/117)	74.3 (63.4/11.0)	13 271 (11 672/1599)	71.2 (62.6/8.6)	
Procedure	APR	189	92.6	1021	95.7	17 873	95.8	0.090
	TPE	15	7.4	46	4.3	778	4.2	
General anesthesia time (min)*		568	436–748	464	373–583	448	359–562	< 0.001
Length of stay (days)*		30.0	22.8–45.0	26.0	19.0–37.0	26.0	19.0–37.0	< 0.001

Abbreviations: APR, abdominoperineal resection; TPE, total pelvic exenteration.

* Median, interquartile range.

### 3.3 Postoperative Complications

Postoperative complications are listed in Table 3. When comparing patients with anal fistula cancer, anal canal cancer, and rectal cancer, there were significant differences in the incidence rates of wound dehiscence (9.8% vs. 5.2% vs. 4.0%, *p* < 0.001) and UTI (5.4% vs. 7.7% vs. 5.6%, *p* = 0.022). There were no significant differences among the three groups in terms of other complications or postoperative mortality. No significant differences were observed among the three groups; however, in patients with anal fistula cancer, the rates of wound infection (18.6% vs. 15.4% vs. 13.9%, *p* = 0.062) and sepsis (3.9% vs. 1.6% vs. 2.2%, *p* = 0.095) were slightly increased.

**TABLE 3:** Postoperative complications.

	Anal fistula cancer		Anal canal cancer		Rectal cancer		*p*
	*n* = 204		*n* = 1067		*n* = 18 651		
	*n*	%	*n*	%	*n*	%	
Surgical site infection	38	18.6	164	15.4	2586	13.9	0.062
Wound dehiscence	20	9.8	55	5.2	742	4.0	< 0.001
Ileus/Intestinal obstruction	20	9.8	128	12.0	2076	11.1	0.572
Peritonitis	13	6.4	51	4.8	872	4.7	0.468
Urinary dysfunction	14	6.9	80	7.5	1412	7.6	0.975
Urinary tract infection	11	5.4	82	7.7	1048	5.6	0.022
Sepsis	8	3.9	17	1.6	414	2.2	0.095
Pneumonia	7	3.4	37	3.5	862	4.6	0.177
Deep venous thrombosis	4	2.0	15	1.4	323	1.7	0.696
Cerebrovascular disease	3	1.5	18	1.7	273	1.5	0.781
Bleeding	3	1.5	15	1.4	195	1.0	0.350
Acute renal failure	2	1.0	5	0.5	141	0.8	0.476
Pulmonary thrombosis	1	0.5	7	0.7	89	0.5	0.490
Acute coronary syndrome	1	0.5	3	0.3	129	0.7	0.250
Death	2	1.0	15	1.4	172	0.9	0.235

### 3.4 Medical Costs

The medical costs were 2, 328, 100 yen (IQR: 1932550‐3 124 760) yen for anal fistula cancer, 2 033 690 yen (IQR: 1810910‐2 497 890) yen for anal canal cancer, and 2 049 000 yen (IQR: 1819085‐2 488 660) yen for rectal cancer, which showed a significant difference among the three groups (*p* < 0.001); the median costs were highest in patients with anal fistula cancer (Table 4).

**TABLE 4:** Medical costs.

	Anal fistula cancer				Anal canal cancer				Rectal cancer				
	*n* = 204				*n* = 1067				*n* = 18 651				*p*
	Median	IQR			Median	IQR			Median	IQR			
Hospitalization medical costs (yen)	2 328 100	1 932 550	—	3 124 760	2 033 690	1 810 910	—	2 497 890	2 049 000	1 819 085	—	2 488 660	< 0.001

### 3.5 Sensitivity Analysis

Because there were > 5% missing TNM staging data for anal fistula cancer, a sensitivity analysis was conducted for T4, N+, and M1 to better understand the characteristics of these tumors. There were very few missing values for factors other than the TNM classification. More missing values were observed in patients with anal fistula cancer than in those with other cancers in the TNM classification. Thus, the missing values in the TNM classification were deemed to be systematic rather than random. Three types of scenarios were adopted: a scenario excluding missing data, a best‐case scenario in which TX was treated as T3 or lower, NX as N0, and MX as M0, and a worst‐case scenario in which TX was regarded as T4, NX as N+, and MX as M1. The proportion of T4 was 25.0%–40.2% for anal fistula cancer, 23.7%–28.8% for anal canal cancer, and 23.2%–27.3% for rectal cancer, with anal fistula cancer having the highest proportion. The proportion of *N*+ was 17.2%–27.5% for anal fistula cancer, 41.4%–45.7% for anal canal cancer, and 47.2%–51.0% for rectal cancer, with anal fistula cancer having the lowest proportion. The proportion of M1 was 2.5%–11.8% for anal fistula cancer, 9.8%–13.5% for anal canal cancer, and 11.7%–15.5% for rectal cancer, with anal fistula cancer having the lowest proportion. The missing values were < 1% for items other than the TNM classification.

### 3.6 Subgroup Analysis

The TNM classification of patients with anal fistula cancer based on Crohn's Disease is shown in Table 5, and the sensitivity analysis was conducted. The proportion of T4 was 34.9%–54.0% for anal fistula cancer with Crohn's Disease, and 20.6%–34.0% for anal fistula cancer without Crohn's Disease, with anal fistula cancer with Crohn's Disease having the higher proportion. The proportion of *N*+ was 17.5%–30.2% for anal fistula cancer with Crohn's Disease, and 17.0%–26.2% for anal fistula cancer without Crohn's Disease, with a similar proportion. The proportion of M1 was 3.2%–15.9% for anal fistula cancer with Crohn's Disease, and 2.1%–9.9% for anal fistula cancer without Crohn's Disease, with a similar proportion.

**TABLE 5:** TNM classification of patients with anal fistula cancer based on Crohn's disease.

		With crohn's disease		Without crohn's disease	
		*n* = 63		*n* = 141	
		*n*	%	*n*	%
cT	T0,1	6	9.5	10	7.1
	T2	5	7.9	27	19.1
	T3	18	28.6	56	39.7
	T4	22	34.9	29	20.6
	TX	12	19	19	13.5
cN	N0	44	69.8	104	73.8
	N+	11	17.5	24	17
	NX	8	12.7	13	9.2
cM	M0	53	84.1	127	90.1
	M1	2	3.2	3	2.1
	MX	8	12.7	11	7.8

## 4 Discussion

In this study, the DPC database was used to clarify the clinical characteristics of patients with anal fistula cancer who underwent abdominoperineal resection or total pelvic exenteration. Compared to patients with anal canal or rectal cancer, the background characteristics of patients with anal fistula cancer included a higher proportion of males, younger age, and more smokers. Their tumor‐related characteristics included more advanced local progression and lower frequencies of lymph node and distant metastases. Surgical characteristics included a higher rate of open surgery, a higher proportion of total pelvic exenterations, and longer postoperative hospitalization. Regarding postoperative complications, there was a higher incidence of surgical site infections, wound dehiscence, and sepsis, and higher medical costs.

The characteristics of patients with anal fistula cancer, such as being predominantly male, younger, and more likely to smoke, were similar to those of patients with Crohn's Disease [18, 19, 20]. This similarity is believed to be influenced by the fact that anal fistulas, which can be a source of cancer, are frequently associated with Crohn's disease.

Among the oncological characteristics, the strong tendency for local progression is thought to be due to anal fistula cancer arising from the fistula tract. To our knowledge, no studies have directly compared the degree of local invasion between anal fistula cancer and rectal cancer; however, strong local invasiveness of anal fistula cancer has been reported, and the need for intensive local treatment has been noted [7, 10]. Similarly, studies comparing anal canal cancer and anal cancer, including cancer arising from anal fistulas, with rectal cancer have reported that these cancers tend to exhibit stronger local invasiveness than rectal cancer [8, 21]. Anal fistula cancer is more likely to spread to the surrounding tissues from the outset than anal canal or rectal cancers, which originate from the intestinal mucosa. Furthermore, pronounced local progression leads to a higher frequency of total pelvic exenteration. As the extent of resection in the buttock region increases, the incidence of incisional wound infections and perineal wound dehiscence is thought to increase. These increases have resulted in an increased incidence of sepsis, longer hospital stays, and higher costs.

No reports have directly compared the frequency of lymph node metastasis (LNM) between patients with anal fistulas and rectal cancers. However, compared to rectal cancer, anal and anal canal cancers are reported to have a lower frequency of mesenteric LNM and a higher frequency of inguinal LNM [7, 8, 10, 21, 22]. In this study, there was no detailed information about lymph node dissection; however, for the three types of cancers, mesenteric lymph node dissection was generally performed, whereas inguinal lymph node dissection was not generally performed as a routine procedure, except in cases where inguinal LNM was suspected preoperatively based on imaging or other examinations. Therefore, inguinal LNM may have been overlooked during surgery. Thus, it is more appropriate to understand that the low frequency of LNM in anal fistula cancer applies only to mesenteric LNM. Furthermore, because distant metastasis was also less common, it is thought that, as a characteristic of anal fistula cancer, it has a lower tendency to metastasize than the other two cancers.

In this study, anal fistula cancer demonstrated stronger local invasiveness than other cancers; however, there was no higher frequency of lymph node or distant metastases. Previous reports have indicated that anal fistula cancer is often diagnosed at an advanced stage, and our findings regarding the T factor are consistent with these reports [23]. However, our results differed from those of previous reports with respect to the N and M factors. Previous reports recommend imaging studies when symptoms worsen. In contrast, in Japan, because anal fistula cancer is often preceded by conditions such as anal fistulas or Crohn's Disease, periodic imaging studies are often conducted, even though there are no fixed guidelines. This may be one of the reasons why, in this study, anal fistula cancer did not show an increase in lymph node or distant metastases compared to other cancers. Additionally, our study used clinical TNM classification. In particular, regarding lymph node metastasis, there can be discrepancies between clinical and pathological staging; therefore, it is possible that our study underestimated the incidence of lymph node metastasis.

There are differences in anal fistula cancer depending on whether it is associated with Crohn's Disease. Anal fistula cancers associated with Crohn's Disease are often accompanied by mucinous components and tend to show strong local invasiveness [4, 10]. Therefore, in this study, a subgroup analysis was conducted to evaluate the TNM classification of anal fistula cancer according to the presence or absence of Crohn's Disease. In anal fistula cancer, no differences were observed in lymph nodes or distant metastases based on the presence of Crohn's Disease; however, stronger local invasiveness was found in anal fistula cancer coexisting with Crohn's Disease.

This study used large‐scale DPC data, including claims and clinical information. As a result, it was possible to clarify the characteristics of rare cancers, such as anal fistula cancer. The results of this study may serve as valuable information for epidemiological investigations of anal fistula cancer and medical policy. However, this study has some limitations. As it is based on DPC data, the available clinical information is limited. Details of the surgeries, pathological findings, and surgeon information were not included. Pathological information, including histological subtypes and margin status, could not be assessed; therefore, the clinical TNM classification was used. As discrepancies are often observed between clinical and pathological diagnoses, the absence of pathological information may have influenced the results of this study. The DPC data used in this study comprised information collected from various medical institutions. Although the characteristics of these institutions may influence the choice of surgical methods and short‐term outcomes, this has not been considered in this study [24]. However, because surgeries for anal fistula cancer are often performed at large hospitals, it can be considered that the data adequately reflect the current state of anal fistula cancer treatment in Japan.

In conclusion, compared to anal canal cancer and rectal cancer, anal fistula cancer was characterized by a more aggressive local progression, resulting in a higher frequency of open surgery and total pelvic exenteration, a higher incidence of wound‐related complications, and higher medical costs.

## Author Contributions

**Nobuaki Hoshino:** conceptualization, methodology, data curation, formal analysis, writing – original draft. **Hiromitsu Kinoshita:** methodology, data curation, writing – review and editing. **Kazutaka Obama:** supervision, writing – review and editing. **Susumu Kunisawa:** methodology, supervision, writing – review and editing, data curation. **Kiyohide Fushimi:** project administration, writing – review and editing, supervision, resources. **Yuichi Imanaka:** project administration, writing – review and editing, supervision.

## Funding

This work was supported by Health and Labor Sciences Research Grant, 24AA2006. JSPS KAKENHI, 23H00448.

## Ethics Statement

Approval of the Research Protocol by an Institutional Review Board: This study was approved by the Ethics Committee of Kyoto University (ID: R0135).

## Consent

The need for informed consent was waived because of the anonymity of the study data; however, the opportunity to withdraw consent by opting out was guaranteed.

## Conflicts of Interest

The authors declare no conflicts of interest.

## Supporting information


**Table S1:** Supporting Information.
Table S2: Supporting Information.

## Data Availability

The data that support the findings of this study are available from the corresponding author upon reasonable request.
