# Supplementary material for: Characteristics of Anal Fistula Cancer in Patients Who Underwent Surgery: A Nationwide Observational Study Using Diagnosis Procedure Combination Data
Source: Ann Gastroenterol Surg. 2026 May 5:10.1002/ags3.70228. Online ahead of print. doi: 10.1002/ags3.70228 (PMC13394022; doi:10.1002/ags3.70228)
Supplement: Supplementary file 1 — Table S1: Supporting Information. Table S2: Supporting Information. [file AGS3-9999-0-s001.docx]

| Table S1 | | | | | | | | | | | |
| --- | --- | --- | --- | --- | --- | --- | --- | --- | --- | --- | --- |
| Comorbidity | ICD-10 code | | | | | | | | | | |
| Hypertension | I10 | I110 | I119 | I120 | I129 | I139 | I150 | I151 | I152 | I159 | I674 |
| Diabetes | E10 | E100 | E101 | E102 | E103 | E104 | E105 | E106 | E11 | E110 | E111 |
|  | E112 | E113 | E114 | E115 | E116 | E12 | E13 | E14 | E140 | E141 | E142 |
|  | E143 | E144 | E145 | E146 | E148 | E149 | E881 | E891 | R730 |  |  |
| COPD | J431 | J432 | J438 | J439 | J440 | J441 | J448 | J449 | J983 | P250 |  |
| Angina | I200 | I201 | I208 | I209 |  |  |  |  |  |  |  |
| Myocardial infarction | I210 | I211 | I212 | I213 | I214 | I219 | I220 | I221 | I228 | I229 | I230 |
|  | I231 | I232 | I233 | I234 | I235 | I236 | I241 | I252 |  |  |  |
| Cerebral infarction | G819 | I630 | I631 | I632 | I633 | I634 | I635 | I636 | I638 | I639 | I693 |
| Cerebral bleeding | I614 | I619 | I691 |  |  |  |  |  |  |  |  |
| COPD: chronic obstructive pulmonary disease | | | | | | | | | | | |

| Table S2 | | | | | | | | | | | |
| --- | --- | --- | --- | --- | --- | --- | --- | --- | --- | --- | --- |
| Complication | ICD-10 code | | | | | | | | | | |
| Surgical site infection | K650 | N735 | T793 | T813 | T814 |  |  |  |  |  |  |
| Wound dehiscence | T813 |  |  |  |  |  |  |  |  |  |  |
| Pneumonia | J120 | J121 | J122 | J123 | J128 | J129 | J13 | J14 | J150 | J151 | J152 |
|  | J153 | J154 | J155 | J156 | J157 | J158 | J159 | J160 | J17 | J180 | J181 |
|  | J182 | J188 | J189 | J690 | J691 | J958 |  |  |  |  |  |
| Pulmonary embolism | I260 | I269 |  |  |  |  |  |  |  |  |  |
| Ileus and bowel obstruction | K560 | K561 | K562 | K563 | K564 | K565 | K567 | K913 |  |  |  |
| Acute coronary syndrome | I210 | I211 | I212 | I213 | I214 | I219 | I220 | I221 | I228 | I229 | I230 |
|  | I231 | I232 | I233 | I234 | I235 | I236 | I238 | I240 | I241 | I248 | I249 |
|  | I251 | I252 | I253 | I254 | I255 | I256 | I258 | I259 |  |  |  |
| Cerebrovascular disease | I600 | I601 | I602 | I603 | I604 | I605 | I606 | I607 | I608 | I609 | I610 |
|  | I611 | I613 | I614 | I615 | I616 | I618 | I619 | I620 | I621 | I629 | I630 |
|  | I631 | I632 | I633 | I634 | I635 | I636 | I638 | I639 | I64 | I650 | I651 |
|  | I652 | I653 | I660 | I661 | I662 | I663 | I668 | I669 |  |  |  |
| Acute renal failure | N170 | N171 | N172 | N178 | N179 |  |  |  |  |  |  |
| Urinary tract infection | N10 | N300 | N301 | N302 | N303 | N304 | N308 | N309 | N340 | N341 | N342 |
|  | N343 | N390 | N391 | N392 | N393 | N394 | N399 |  |  |  |  |
| Sepsis | A021 | A227 | A241 | A267 | A282 | A327 | A394 | A400 | A401 | A402 | A403 |
|  | A408 | A409 | A410 | A411 | A412 | A413 | A414 | A415 | A418 | A419 | A548 |
|  | B007 | B349 | B377 |  |  |  |  |  |  |  |  |
| Dysuria | N310 | N311 | N312 | N318 | N319 | N990 | N991 | N992 | N993 | N994 | N998 |
| Venous thrombosis | I802 | I803 |  |  |  |  |  |  |  |  |  |
| Peritonitis | K650 | K658 | K659 | N735 |  |  |  |  |  |  |  |
| Intestinal ischemia | K550 | K551 | K559 |  |  |  |  |  |  |  |  |
| Bleeding | K661 | T810 |  |  |  |  |  |  |  |  |  |
|  |  |  |  |  |  |  |  |  |  |  |  |
